# Plasma Alkylresorcinols Is an Objective Biomarker for Gluten Intake in Young Children

**DOI:** 10.1016/j.tjnut.2025.01.020

**Published:** 2025-01-27

**Authors:** Elin M Hård af Segerstad, Emelie Ericson-Hallström, Anna Bokström, Marina Armeni, Otto Savolainen, Carin Andrén Aronsson

**Affiliations:** 1 Pediatric Research Institute, Oslo University Hospital, Oslo, Norway; 2 Department of Clinical Sciences, Lund University, Malmö, Sweden; 3 Department of Pediatrics, Skåne University Hospital, Malmö, Sweden; 4 Chalmers Mass Spectrometry Infrastructure, Department of Life Sciences, Chalmers University of Technology, Gothenburg, Sweden; 5 Institute of Public Health and Clinical Nutrition, University of Eastern Finland, Kuopio, Finland

**Keywords:** alkylresorcinol, gluten, biomarker, children, nonfasting, clinical trial

## Abstract

**Background:**

Alkylresorcinols are a well-established biomarker for whole-grain intake. There is evidence suggesting that total plasma alkylresorcinol concentration may also be used as a biomarker for gluten intake in adults.

**Objectives:**

The aim of this study was to evaluate if total alkylresorcinol concentration is a valid biomarker for gluten intake in young children.

**Methods:**

Nonfasting plasma alkylresorcinol concentrations were analyzed by normal-phase ultrahigh-pressure liquid chromatography-tandem mass spectrometry in 65 children aged 18 mo included in a randomized controlled trial. The intervention group was following a gluten-free diet (*n* = 21, 31.3%), whereas the diet was unrestricted in the control group (*n* = 44, 65.7%). Alkylresorcinol concentrations in the 65 children were validated against simultaneously collected 3-d food records estimating total gluten intake.

**Results:**

Gluten intake in controls was median 5.8 grams/d (IQR: 2.8–9.4, max 17.1) compared with 0.0 g/d (IQR: 0.0–0.0, max 0.7, *P* < 0.001) in the intervention group. In the control group, wheat accounted for mean 85% (SD: 0.1) of the gluten intake. The intervention group had lower alkylresorcinol levels (median: 7.2 nmol/L; IQR: 4.0–10.5) compared with controls (median: 269; IQR: 116–505 nmol/L, *P* < 0.001). The correlation between alkylresorcinol concentrations and gluten intake was ρ = 0.68 (*P* < 0.001). Alkylresorcinol concentrations increased by 35.7% [95% confidence interval (CI): 25.9, 46.2, *P* < 0.001] for every g/d increase of gluten intake. The Cohen’s weighted kappa between quartiles of alkylresorcinol and gluten intake was 0.73 (95% CI: 0.59, 0.86).

**Conclusions:**

Alkylresorcinol concentrations increased with gluten intake in young nonfasting children. The findings suggest that alkylresorcinol concentrations may be a useful biomarker for gluten intake in young children.

This trial was registered at clinicaltrials.gov as NCT03562221.

## Introduction

Alkylresorcinols (ARs) are a well-established biomarker validated to reflect whole-grain wheat and rye intake in adults, adolescents, and children as young as 8 y old [[1], [2], [3], [4], [5]]. Although ARs have a short half-time, they have been shown to be reliable for habitual intake over time [6,7].

Even if the concentration of ARs in refined grains is lower than that in their whole-grain counterparts, blood plasma samples will have measurable AR concentrations [8,9]. In addition to being a biomarker of whole-grain intake, there is evidence that AR may be used as a biomarker of dietary gluten intake in adults [10,11]. Gluten includes various proteins found in the gluten-containing grains wheat, rye, and barley, a complex mixture of storage proteins (gliadin and glutenin) rich in proline and glutamine amino acids and resistant to complete degradation by enzymes in the small intestine [12]. In celiac disease, a chronic immune-mediated intolerance to gluten, there is a need to objectively assess gluten intake in both research and clinical settings, whether to estimate gluten amounts before the onset of celiac disease [[13], [14], [15]] or as a measure of adherence to a gluten-free diet [16].

Nutritional research traditionally relies on self-reported dietary intake data to estimate true intakes. The 3- or 7-d food record method is considered to be the gold standard as it is a prospective assessment method, in contrast to retrospective methods such as the food frequency questionnaire (FFQ) [17]. However, misreporting of dietary intake is common and inconsistent across different foods and populations, leading to biased data that may obscure true associations between diet and health outcomes [17,18]. Biomarkers that reflect nutrient and food intake are objective markers that help overcome measurement errors and reporting biases, although providing a complement to traditional dietary assessment methods [19].

The aim of the study was to evaluate whether total plasma AR is a valid biomarker of gluten intake in young children at genetic risk for celiac disease by comparing it with estimates from a 3-d food record.

## Methods

### Study population

We used data from the ongoing intervention study “Prevention of Celiac disease in Skåne” (PreCiSe) in the south of Sweden. The study enrolls infants screened at birth for the celiac disease-associated human leucocyte antigen genotype DR3-DQ2/DR3-DQ2 [20]. The eligible infants (1.5% of the screened population), were invited and randomly assigned to follow either a strict gluten-free diet or an unrestricted diet from age 4 mo to age 3 y [21].

In this study, we included 65 children (gluten-free diet *n* = 21, unrestricted diet *n* = 44) who, in accordance with the study protocol, provided a nonfasting plasma sample and a concurrent 3-d food record at their 18-mo study visit, collected between February 2020 and June 2022. The PreCiSe study is registered in ClinicalTrials.gov Identifier NCT03562221 and approved by the Swedish Ethical Review Authority. Caregivers gave informed consent for both to the initial screening and the intervention study.

### Alkylresorcinol analysis

Plasma samples were separated by centrifugation (2000G) and aliquoted in microtubes (200 μL), which were stored at –80°C until analyses. From the collected plasma samples, AR concentrations were analyzed at Chalmers Mass Spectrometry Infrastructure by using normal-phase ultrahigh-pressure liquid chromatography-tandem mass spectrometry. This method has previously been described in detail elsewhere [22]. In brief, 100 μL of plasma was loaded on HybridSPEPlus phospholipid removal 96-well plates (Sigma-Aldrich) followed by the addition of stable isotope-labeled internal standard (nonadecylresorcinol-d4, C19:0 d4 (ReseaChem GmbH) and recovered with acetone. After evaporating and resuspending the eluted samples in heptane-ethanol (95:5 volume/volume), extracts were transferred to chromatographic vials and analyzed by liquid chromatography-tandem mass spectrometry (QTRAP 6500+, AB SCIEX). Analytes were separated using a 50 × 2.1 mm amino column with 1.8-mm particles (Hypersil GOLD, Thermo Fischer Scientific) using a gradient program with solvents A and B, heptane, and ethanol, respectively. The liquid chromatography column was kept at 30°C. Ionization was conducted using atmospheric pressure chemical ionization in positive mode. Optimal conditions for the multiple reaction monitoring mode were set for individual AR homologues. Data were processed with MultiQuant software. Concentrations were defined for AR homologues C17:0, C19:0, C21:0, C23:0, and C25:0, as well as their sum as total AR concentration. In addition, the ratio of C17:0/C21:0 was calculated. The C17:0 to C21:0 ratio is used to determine the primary source of AR in the diet, with values of 1.0 reflecting rye, 0.1 wheat, and 0.01 durum wheat [2].

### Gluten intake assessment

Primary caregivers were instructed to keep a 3-d food record of the child’s dietary intake for 2 consecutive weekdays and 1 weekend day within a week before the study visit. Portion sizes were estimated using either standard measuring cups and containers (for example, bowls, cups, and glasses), a household scale, or food portion photographs of common foods and food shapes. This method has previously been used in a large multinational cohort study [23]. The collected food records were reviewed and probed for missing information at the study visit by a research nurse followed by a research dietitian. The research dietitians entered the reported foods and drinks into the computerized calculation program NutriDb (BC Platforms). Food and nutrient intake were calculated using the Swedish food composition database provided by the Swedish National Food Administration, combined with a local food database containing additional foods and drinks, specifically commercial infant foods. The mean daily intakes of wheat, rye, and barley were summarized. The mean daily gluten intake was estimated based on the total amount of protein in gluten-containing grains and by applying a conversion factor of 0.8 for wheat, 0.65 for rye, and 0.5 for barley [24,25].

### Assessment of adherence to the gluten-free diet

Research dietitians assessed the adherence to the gluten-free diet in the intervention group using 2 methods: reviewing the collected 3-d food records for reported gluten intake, and conducting a systematic interview with the caregivers at the child’s annual visits, where dietary adherence was categorized as described elsewhere [26]. At 1 and 2 y of age, all children in the intervention group were assessed as having excellent adherence to the gluten-free diet (age 1 y: *n* = 3 missing, age 2 y: *n* = 3 missing, *n* = 1 withdrawn).

### Statistical methods

The correlation between total AR, AR homologues, and estimated gluten intake (absolute amounts and energy-adjusted amounts standardized to per 1000 kcal intake [27]), total gluten-containing grains, wheat, rye, barley, and dietary fiber (as a proxy for whole-grain intake) were tested by using Spearman’s rank correlation analysis in the full cohort of 65 children. By visual inspection of a boxplot, we identified 2 outliers in AR levels (>1000 nmol/L). After removing the 2 outliers and log-transforming AR levels, the assumptions were met to perform a linear regression to analyze the association between gluten intake in g/d with total AR levels (dependent variable). Including sex in the model did not improve the model fit and was, therefore, not included as a covariate. In addition, total AR and gluten intake (g/d) were categorized in quartiles, and Cohen’s kappa (K) was used to determine the level of agreement and misclassification, as well as grossly misclassified subjects (that is, placed in opposite quartile).

Descriptive population data are expressed as means with SD or median with IQR, depending on the distribution of the data. The Mann–Whitney *U* test and χ^2^ test were used to identify potential differences between the intervention and control group and for within-group comparisons. A *P* < 0.05 was considered to be statistically significant. Statistical analyses were performed in IBM SPSS Statistics for Windows, version 29.0 (IBM Corp., 2022), and GraphPad Prism 10.3.1.

## Results

**FIGURE 1:**
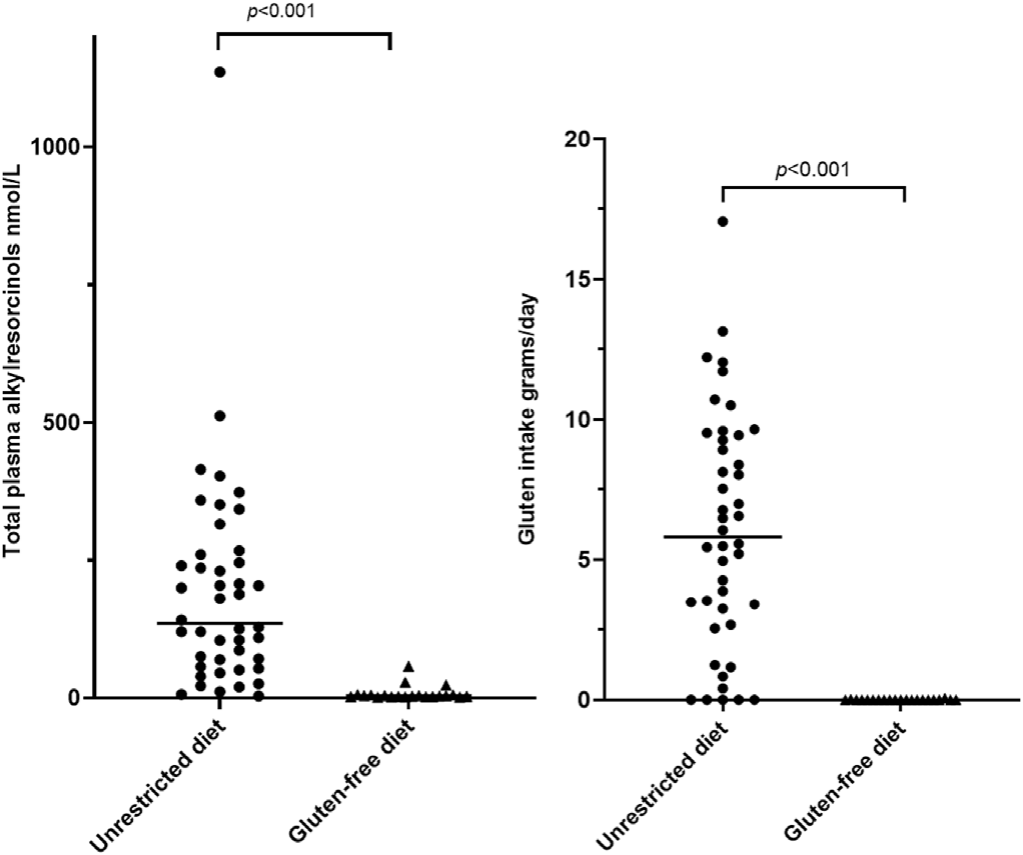
Nonfasting total plasma alkylresorcinols and gluten intake in young children participating in a dietary intervention study by the intervention group (unrestricted diet *n* = 44 and gluten-free diet *n* = 21).

The intervention and control groups were similar in age at the time of data collection, as well as in the distribution of sex, body weight, and the number of children having a family member with celiac disease (Table 1). The intake of total energy, carbohydrates, dietary fiber, proteins, and total fat were also similar across the 2 groups. The median intake of total gluten was 0.0 (IQR: 0.0–0.0) g/d in the intervention group and 5.8 (IQR: 2.8–9.4) g/d in the control group (*P* < 0.001, Figure 1). The control group had higher intakes of wheat, rye, barley, and oats compared with the intervention group (*P* < 0.001, <0.001, 0.039, and 0.023, respectively).

**TABLE 1:** Descriptive and dietary data in 65 Swedish children participating in a dietary intervention study, at age 18 mo.

*Descriptive data*	Control group unrestricted diet (*n* = 44)		Intervention group gluten-free diet (*n* = 21)		Between group *P* value
		Within group *P* value		Within group *P* value	
Age at sampling (mo), median (IQR)	18.1 (18.0–18.4)	–	18.1 (17.9–18.5)	–	0.784
Females	18.2 (18.0–18.4)	0.972	18.3 (18.1–19.6)	0.129	–
Males	18.1 (18.0–18.5)		18.1 (17.8–18.5)		–
Female sex, *n* (%)	24 (54.5)	–	9 (42.9)	–	0.378
Body weight (kg), mean (SD)	12.1 (1.5)	–	12.6 (1.7)	–	0.280
Females	11.6 (1.5)	0.038	11.9 (10.6–13.1)	0.148	–
Males	12.5 (1.3)		12.7 (11.9–14.1)		–
Body mass index, median (IQR)	17.2 (16.6, 18.3)	–	17.6 (16.8, 19.0)	–	0.393
Females	16.8 (16.3, 18.2)	0.164	16.8 (15.6, 19.1)	0.272	–
Males	17.5 (16.8, 18.4)		18.0 (17.1, 19.0)		–
Family member with celiac disease, *n* (%)	13 (29.5)	–	3 (14.3)	–	0.182
*Dietary intake g/d, median (IQR)*					
Gluten,	5.8 (2.8–9.4	–	0.0 (0.0–0.0)	–	<0.001
Females	5.5 (2.8–7.4)	0.258	0.0 (0.0–0.0)	0.972	–
Males	8.1 (2.8–9.6)		0.0 (0.0–0.0)		–
Gluten, per 1000 kcal	5.7 (2.5–8.7)	–	0.0 (0.0–0.0)	–	<0.001
Females	5.4 (2.5–8.7)	0.741	0.0 (0.0–0.0)	0.972	–
Males	6.4 (2.4–8.7)		0.0 (0.0–0.0)		–
Wheat	55.7 (25.0–86.6)	–	0.0 (0.0–0.0)	–	<0.001
Females	50.7 (19.5–63.1)	0.195	0.0 (0.0–0.0)	0.972	–
Males	71.7 (27.7–93.0)		0.0 (0.0–0.0)		–
Rye	6.2 (0.1–18.5)	–	0.0 (0.0–0.0)	–	<0.001
Females	3.6 (0.0–11.2)	0.096	0.0 (0.0–0.0)	1.000	–
Males	10.2 (2.8–22.2)		0.0 (0.0–0.0)		–
Barley	0.0 (0.0–0.0)	–	0.0 (0.0–0.0)	–	0.039
Females	0.0 (0.0–0.0)	0.889	0.0 (0.0–0.0)	1.000	–
Males	0.0 (0.0–0.0)		0.0 (0.0–0.0)		–
Oats	18.7 (0.1–60.9)	–	4.8 (0.1–9.6)	–	0.023
Females	7.8 (0.0–60.8)	0.142	2.5 (0.0–11.1)	0.754	–
Males	25.8 (4.5–67.4)		4.8 (1.5–7.9)		–
*Macronutrients, median (IQR)*					
Total energy intake, kcal/d	993 (936–1124)	–	1009 (886–1107)	–	0.623
Females	963 (834–1050)	0.003	940 (852–1128)	0.508	–
Males	1119 (960–1219)		1029 (919–1118)		–
Carbohydrates (g/d)	123 (98.8–139)	–	122 (111–133)	–	0.768
Female	106 (86.3–137)	0.003	121 (108–136)	0.508	–
Male	130 (122–150)		127 (113–133)		–
Carbohydrates, % of energy	47.0 (43, 52)	–	49.9 (47.3, 53.4)		0.048
Females	48.4 (42.9, 51.4)	0.282	49.2 (48.4, 51.1)	0.651	–
Males	48.9 (44.4, 52.7)		52.4 (43.3, 55.9)		–
Protein (g/d)	35.4 (30.0–42.6)	–	32.9 (29.4–36.0)	–	0.087
Females	34.4 (28.1–41.5)	0.138	32.9 (30.1–36.0)	0.917	–
Males	37.9 (31.4–43.6)		32.8 (27.5–37.5)		–
Proteins, % of energy	14.1 (12.3, 15.3)	–	12.8 (11.2, 14.5)		0.119
Females	13.6 (12.5, 15.7)	0.055	13.8 (11.6, 15.1)	0.422	–
Males	12.8, 11.4, 14.5)		12.6 (11.2, 13.8)		–
Fat (g/d)	39.2 (34.0–48.5)	–	35.3 (31.1–48.6)	–	0.333
Females	34.7 (32.2–45.5)	0.001	33.8 (31.1–45.3)	0.651	–
Males	43.2 (37.5–51.2)		37.0 (30.2–53.0)		–
Fat, % of energy	36.7 (33.3, 40.1)	–	34.9 (30.4, 38.2)		0.145
Females	35.9 (32.6, 39.2)	0.937	33.0 (32.6, 36.6)	0.702	–
Males	35.0 (31.9, 40.4)		32.0 (28.9, 43.8)		–
Dietary fiber (g/d)	10.7 (8.4–13.5)	–	11.4 (9.1–12.4)	–	0.833
Females	9.3 (7.4–14.0)	0.358	10.7 (8.1–12.1)	0.247	–
Males	11.4 (9.2–13.2)		11.8 (9.7–13.5)		–

Abbreviations: g, grams; kg, kilograms.

The Mann–Whitney *U* test and the Chi-square test were used for between and within-group comparisons.

In the control group, males had higher body weight, energy, and carbohydrate and fat intake compared with females (*P* = 0.001–0.038, Table 1). There was a similar trend observed between males and females for body weight, energy, and fat intake in the intervention group, although it did not reach statistical significance. Body mass index was similar between the groups but higher, although not statistically different, in males.

### Plasma alkylresorcinol concentration in participants

The median total AR concentration was 7.2 (IQR: 4.0–10.5) nmol/L in the intervention group compared with 269 (IQR: 116–505) nmol/L in the control group (*P* < 0.001) (Table 2, Figure 1). The difference between the groups was observed across all AR homologues (all *P* < 0.001) but not with the C17:C21 ratio (*P* = 0.877). In the control group, males had levels of AR that were more than twice as high as the females levels (median 430, IQR: 217–703 nmol/L, and median 200, IQR: 98.5–387 nmol, respectively, *P* = 0.022), and differences were observed in all the AR homologues (*P* = 0.007–0.022).

**TABLE 2:** Nonfasting plasma alkylresorcinol concentrations in 65 children, age 18-mo and participating in a dietary intervention study.

	Control group unrestricted diet (*n* = 44) (female *n* = 24)		Intervention group gluten-free diet (*n* = 21) (female *n* = 9)		Between group *P* value
	Median (IQR)	Within group *P* value	Median (IQR)	Within group *P* value	
Alkylresorcinol homologues (nmol/L)					
Total alkylresorcinols	269 (116–505)		7.2 (4.0–10.5)		<0.001
- Female	200 (98.5–387)	0.022	7.8 (3.6–29.2)	0.862	
- Male	431 (217–703)		5.7 (3.9–9.9)		
C17:C21 ratio	0.13 (0.09–0.15)		0.13 (0.07–0.17)		0.877
- Female	0.11 (0.6–0.13)	0.025	0.11 (0.04–0.23)	1.000	
- Male	0.13 (0.10–0.22)		0.13 (0.07–0.17)		
C17	15.1 (6.6–30.3)		0.4 (0.2–0.5)		<0.001
- Female	9.2 (3.1–18.6)	0.008	0.4 (0.3–0.4)	0.972	
- Male	27.2 (9.7–43.6)		0.3 (0.2–0.5)		
C19	77.9 (27.7–142.3)		1.6 (1.0–2.4)		<0.001
- Female	52.4 (24.6–120–5)	0.020	2.1 (0.8–3.8)	0.651	
- Male	121 (70.0–197)		1.4 (1.0–2.3)		
C21	121.8 (53.1–259.2)		3.2 (1.6–5.3)		<0.001
- Female	87.4 (48.6–173.8)	0.022	3.6 (1.3–16.3)	0.808	
- Male	187 (102–331)		2.9 (1.7–4.9)		
C23	39.9 (16.4–72.3)		0.9 (0.5–1.6)		<0.001
- Female	24.5 (14.2–47.8)	0.017	0.9 (0.4–5.2)	0.754	
- Male	64.8 (20.0–99.0)		2.9 (1.7–4.9)		
C25	21.5 (6.9–36.9)		0.6 (0.3–1.0)		<0.001
- Female	10.1 (6.1–22.9)	0.007	0.8 (0.4–1.7)	0.247	
- Male	34.5 (13.6–56.9)		0.5 (0.3–0.9)		

The Mann–Whitney *U* test and the Chi-square test were used for between and within-group comparisons.

**FIGURE 2:**
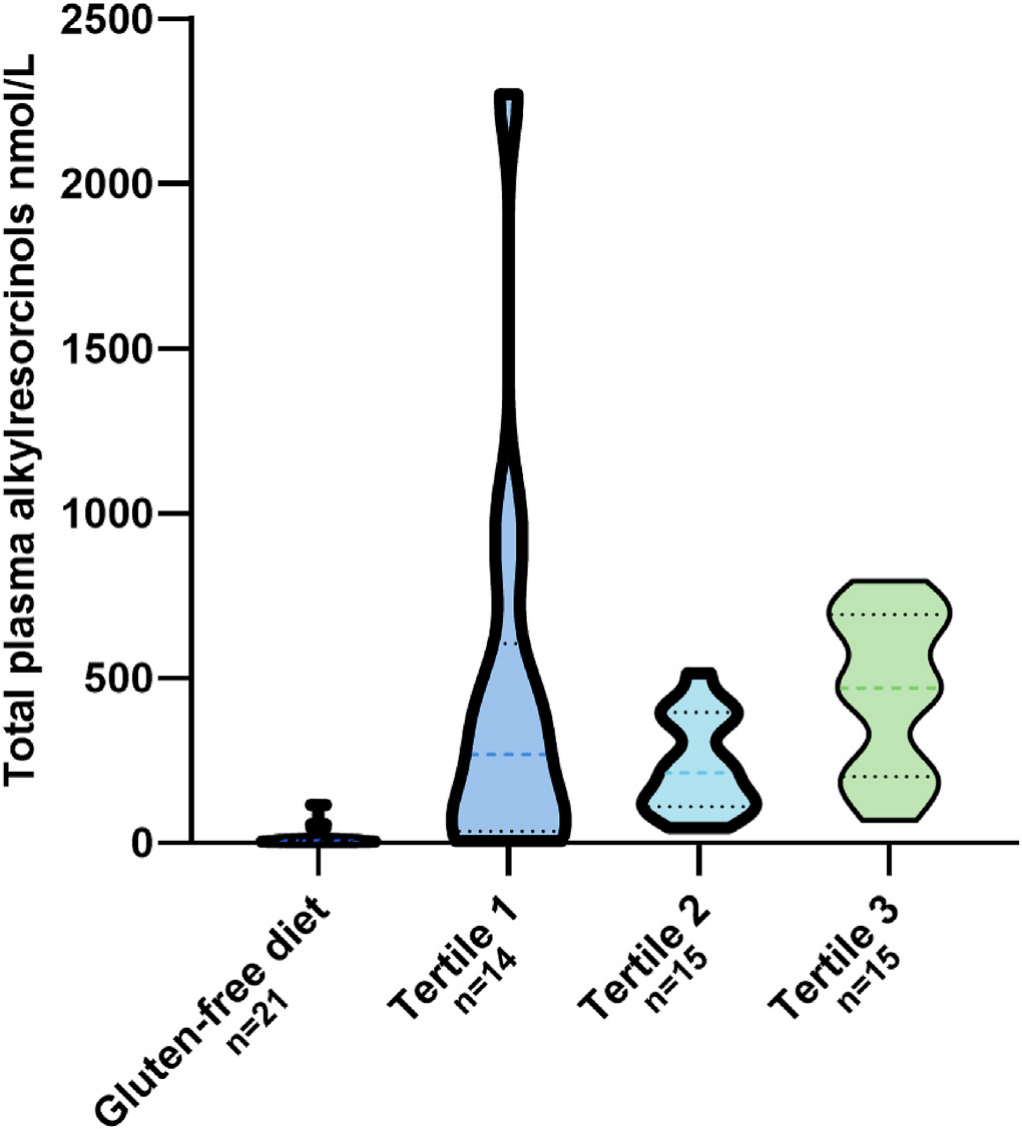
Violin plot of nonfasting total plasma alkylresorcinols and gluten intake by 4 categories; participants following a gluten-free diet, and by gluten intake in tertiles in those with unrestricted gluten intake. Dashed lines represent the median, and dotted lines the 25th and 75th percentiles.

The distribution of total AR by gluten-free diet and gluten intake in tertiles are presented in Figure 2 and Table 3. The median total AR was 269 (IQR: 34.6, 605) in tertile 1 and 212 (IQR: 110, 396) in tertile 2. Of the 2 identified outliers in total AR (both in tertile 1), 1 had the highest concentrations of all homologues, 0.5–3 times higher than the second highest concentrations. The other outlier had high concentrations of C19 and C21, but the homologues C17, C23, and C25 were similar to other nonoutlier observed values.

**TABLE 3:** Nonfasting total plasma alkylresorcinols and gluten intake assessed by 3-d food records in children at age 18 mos by 4 categories; participants following a gluten-free diet, and by gluten intake in tertiles in those with unrestricted gluten intake.

	Gluten-free diet (*n* = 21)	Gluten intake, first tertile (*n* = 14)	Gluten intake, second tertile (*n* = 15)	Gluten intake, third tertile (*n* = 15)
Gluten intake, median (IQR) g/d	0.0 (0.0, 0.0)	1.0 (0.0, 2.8)	5.6 (4.9, 6.8)	9.7 (9.3, 12.0)
Total alkylresorcinol, nmol/L, median (IQR)	7.2 (4.0, 10.5)	269 (34.6, 605)	212 (110, 396)	470 (201, 693)

Abbreviations: g, grams; L, liter.

### Validation of plasma alkylresorcinols against 3-d food records

**FIGURE 3:**
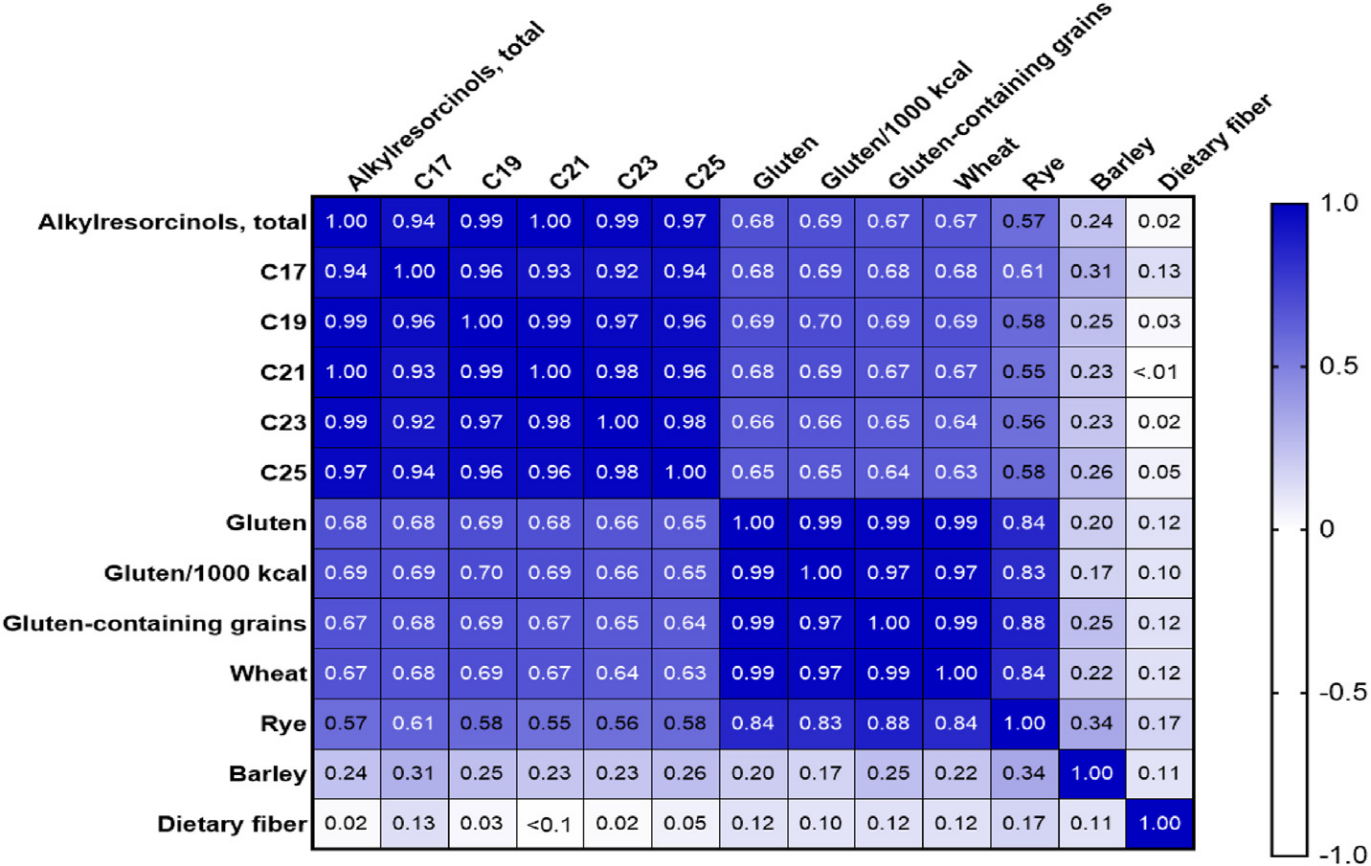
Spearman correlation coefficient matrix between nonfasting total alkylresorcinol and alkylresorcinol homologues, gluten, gluten-containing grains and dietary fiber in 65 children at the age of 18 mo. Dietary intake was estimated by 3-d food records.

**FIGURE 4:**
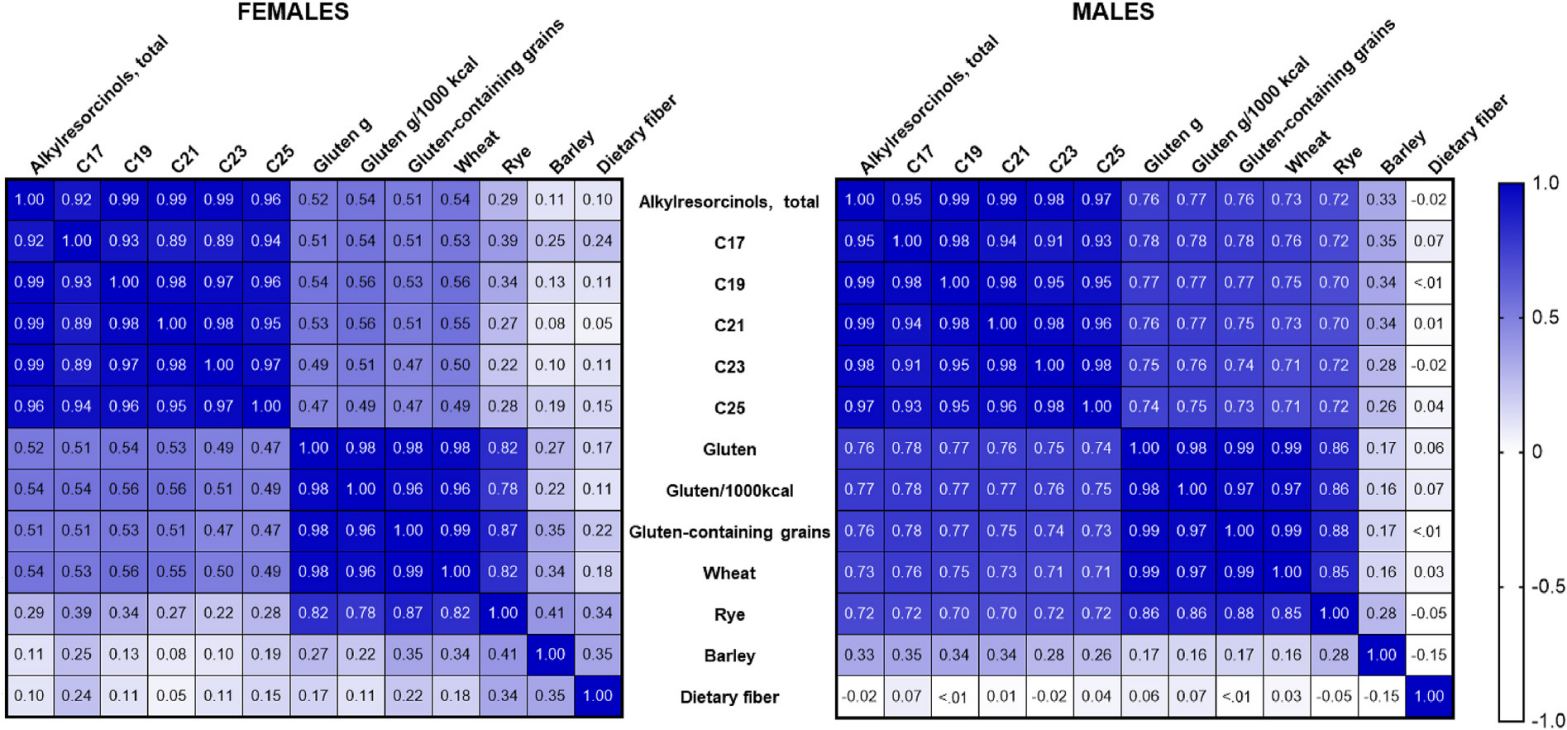
Spearman correlation coefficient matrix by sex between total alkylresorcinol and alkylresorcinol homologues, gluten, gluten-containing grains and dietary fiber in 33 females and 32 males at the age of 18 mo. Dietary intake was estimated by 3-d food records.

The correlation between total AR and daily gluten intake in the full cohort was ρ = 0.68 (*P* < 0.001) and between total AR and gluten intake/1000 kcal ρ = 0.69 (*P* < 0.001, Figure 3, Supplemental Table 1). In comparison, the correlation between AR and total intake of gluten-containing cereals (wheat, rye, and barley) was ρ = 0.67 (*P* < 0.001), wheat intake ρ = 0.67 (*P* < 0.001), rye intake ρ = 0.57 (*P* < 0.001), and barley intake ρ = 0.24 (*P* < 0.001). The correlation between total AR, AR homologues, gluten intake (absolute amounts and per 1000 kcal), and each gluten-containing grain was higher in males compared with that in females (Figure 4, Supplemental Table 1). For each 1-gram increase in gluten/d, the AR level increased by 35.7% (95% confidence interval [CI]: 25.9, 46.2, *P* < 0.001, *R*^2^ = 0.52).

The overall agreement between quartiles of AR and gluten intake was Cohen’s weighted K = 0.73 (95% CI: 0.59, 0.86, Table 4). In the first quartile of AR, there was a 100% agreement (gross misclassification 0%) in the same or adjacent quartile and 81.3% in the fourth quartile (gross misclassification 18.7%). For females, the agreement between AR and gluten intakes was Cohen’s weighted K = 0.63 (95% CI: 0.37, 0.89), and for males, the Cohen’s weighted K was 0.79 (0.66, 0.93).

**TABLE 4:** Classification of participants in correct quartile of gluten intake based on plasma alkylresorcinol concentrations and daily gluten intake assessed by 3-d food records in 65 children at age 18 mo.

	Total alkylresorcinols, quartiles				K^1^
	1 (*n* = 16)	2 (*n* = 16)	3 (*n* = 17)	4 (*n* = 16)	
Total alkylresorcinol, median (IQR) nmol/L	5.1 (3.5–7.7)	45.9 (14.7–92.4)	243 (184–366)	645 (472–780)	0.73 (0.59, 0.86
Total gluten intake, median (IQR) g/d	0.0 (0.0–0.0)	3.2 (0.0, 5.2)	6.5 (3.5–8.2)	9.1 (3.8–10.4)	
Same or adjacent quartile, %	100	93.9	94.7	81.3	
Gross misclassification, %	0.0	6.1	5.3	18.7	

Abbreviations: g, grams; L, liter.

1 Cohen weighted K coefficient with lower and upper confidence intervals.

## Discussion

### Summary of main findings

In this validation study, we found that nonfasting plasma AR concentrations correlated strongly with daily gluten intake in free-living children at the age of 18 mo and that the agreement between AR concentrations and gluten intake in quartiles was excellent. AR concentrations increased in a dose-dependent manner for every gram increase in daily gluten intake.

Wheat was the main source of gluten, and both wheat and rye showed a stronger correlation with AR compared with barley. There was a clear difference in AR between the sexes, with more than twice the concentrations in males compared with in females. In males, the correlation between AR and gluten intake was also stronger compared with females. Males tended to have higher intakes of gluten both in absolute and energy-adjusted amounts, a difference that may have reached statistical significance in a larger cohort. The results indicate that AR concentrations may be used as a biomarker for gluten intake in young children, even in a nonfasting state.

### Comparison with previous findings

In previous studies, AR has been found to be an objective and valid marker with a dose-dependent change in concentration with gluten consumption in fasting adults [10,11,28]. A cross-over study found total AR to be substantially higher with a gluten-rich diet compared with a gluten-poor diet [11]. In addition, 2 studies measured total AR before and after diagnosis of celiac diseases in adults as a marker of the initiation of a gluten-free diet [10,28]. Both studies found a significant decrease in total AR after diagnosis. Compared with those in adults [10,11], AR concentrations were more strongly correlated with gluten intake in children aged 18 mo, showed better agreement in quartiles, and the increase in AR concentration was higher in children for each gram increase in gluten daily. One explanation may be that 1 gram of gluten is a higher amount relative to the young child’s body size. Moreover, the measurement error in estimating the gluten intake may differ between this study and previous publications in adults. Self-reported dietary data are always at risk of reporting bias. The retrospective method of FFQs was used in the studies on adults compared with the prospective method of 3-d food record in this study. Food records are generally considered a superior method for self-reported dietary intake because of the lower prevalence of measurement errors [17]. Correlation coefficients between whole-grain intake and AR concentrations have been reported to range between 0.25 and 0.58, and studies using FFQs showed lower correlations compared with food records [29]. Yet, in this population of young children, the correlation with gluten intake was even stronger.

Another explanation is that AR concentrations in adults are not only influenced by dietary intake, but also by plasma lipid levels and other metabolic factors [6,7,11]. Interestingly, in line with findings in adults when using total AR as a biomarker for whole-grain intake [6], we observed a clear difference in AR concentrations depending on the child’s sex; males had considerably higher AR concentrations compared with females. This may in part be explained by a tendency of higher gluten intake in males compared with that in females, but the amount of fat was also higher in the male’s diet. As ARs are lipophilic compounds, higher dietary fat intake may promote the solubilization of AR and, therefore, its absorption. This may, therefore, further attenuate the stronger correlation between AR concentrations and gluten intake in males compared with females. In adults, this phenomenon has also been suggested to relate to plasma lipid concentrations and differences in absorption and metabolism of AR between the sexes [6,7,11]. In this study, plasma lipids were not analyzed but are correlated with AR concentrations. Although future research is needed to explain the sex differences in total AR in young children, the results from this study suggest that plasma AR concentrations may be a useful objective biomarker when assessing gluten intake in young children where a fasting state is not feasible.

### Limitations and strengths

Although single measurements of AR concentrations have been shown to reliably reflect long-term intake of whole grains in adults [6], previous studies have reported a large interindividual variation [29]. This study was cross-sectional with only one measurement of AR concentrations per child and with no comparable data for the age group. Further investigation with multiple samples over time would, therefore, be needed to estimate the interindividual variation of AR concentrations in young children and the validity to reflect long-term gluten intake. As previously suggested, collecting information on the time since the last meal at plasma sampling may improve the understanding of variation in young children’s AR concentrations [29]. As observed in a previous study, wheat contributed with most of the gluten in the diet of young Swedish children [30]. Infant cereals, bread, and pasta are common foods in the children’s diets and are often served daily, suggesting that the gluten intake in the group may still be fairly stable.

Another limitation of this study was that the even-chained homologues were not analyzed. The AR homologues C20 and C22 reflect dietary intake of the gluten-free grain quinoa and contributes to the total concentration of AR. Moreover, we were not able to extract the intake of quinoa in the participants from the food database. However, empirically, quinoa is not a common gluten-free grain in this age group in Sweden and should thus not have a major impact on the findings.

The main strength of this validation study was that AR concentrations were found to be reliable markers of gluten intake even though samples were collected at a nonfasting state. This is a major advantage as fasting is not always feasible in this young age group. However, fasting may have had an impact on the 2 observed outliers in this study. These outliers, both from the control group, reported low gluten intake in the 3-d food records. The AR data of 2 participants were carefully verified to exclude the possibility of misintegration of the individual AR homologue peaks. When reviewing their food records, we found that the number of days between food intake reporting and the date of blood draw was ≤9 d apart. Although these children might not have had a habitually high-gluten intake, the high AR concentrations suggests that they may have consumed a high-gluten meal shortly before the blood draw.

The strong correlation and agreement between AR concentrations and gluten intake suggest that the 3-d food records used had a low measurement error in estimating gluten intake in the young children. The 3-d food record is a standardized method, the research dietitians entering the records into the in-house data software were highly trained, and gluten content in reported foods was carefully considered. The findings also suggest a low parental reporting bias of consumed foods and drinks.

The AR homologue profile varies in different grains, and the C17:C21 ratio may be used to determine the dietary source of whole grain [2]. Ratios lower than 0.2 in plasma samples indicate that the main source is wheat, whereas higher ratios indicate that whole-grain rye is a primary source. In this study, the C17:C21 ratio confirmed that wheat was the predominant source of AR homologues in the children.

### Implications of the findings

There are several possible settings where AR concentrations may be a valuable biomarker for gluten intake in young children. In epidemiological research, an objective marker for gluten intake would be valuable when investigating early life diet and risk of celiac disease. Higher gluten intake in early life has been associated with the development of celiac disease [13,14,15,31]. Although adequate exposure assessment is essential in epidemiologic research, self-reported dietary intake is costly, is time-consuming, requires high literacy, and imposes a high burden on the participants. Moreover, food composition databases typically do not have information of gluten content in foods, and in previous research, gluten intake was instead estimated using a conversion factor based on the protein content in wheat, rye, and barley [24,25]. A reliable biomarker for gluten intake may be used instead of collecting self-reported dietary data, or as a complement to dietary assessment methods with a lower participant burden, such as the FFQ.

Another implication of the findings in this study is that AR concentrations may be used in dietary intervention studies, including young children to distinguish groups with varying gluten intake levels, as in this study. Finally, AR concentrations have been suggested to be used as an objective marker to monitor compliance with a gluten-free diet in adult patients with celiac disease [10,11]. The findings from this study also support this use in young children.

In conclusion, AR concentrations showed a strong correlation with daily gluten intake assessed by 3-d food records in children aged 18 mo. The findings support the use of AR as a biomarker of gluten intake in young children, with extended areas of use in nutrition research and in clinical settings.

## Author contributions

The authors’ responsibilities were as follows – EHS, CAA: designed the research; all authors: conducted the research and provided essential materials; EHS: performed the statistical analysis; EHS, CAA: wrote the paper; CAA: had primary responsibility for the final content; and all authors: read and approved the final manuscript.

## Data availability

Data described in the manuscript, code book, or analytic code will not be made available because of the restrictions in ethical legislation on personal data in Sweden.

## Funding

The work was supported by the Swedish Coeliac Research Fund, the Albert Påhlsson foundation and the Maggie Stephens foundation.

## Conflict of interest

The authors declare no conflict of interest.
