# Supplementary material for: Plasma Alkylresorcinols Is an Objective Biomarker for Gluten Intake in Young Children
Source: J Nutr. 2025 Jan 27;155(3):985–93. doi: 10.1016/j.tjnut.2025.01.020 (PMC11934242; doi:10.1016/j.tjnut.2025.01.020)
Supplement: multimedia component 1 [file mmc1.docx]

Supplemental material to

**“Plasma Alkylresorcinols is an objective biomarker for gluten intake in young children”**

Elin M Hård af Segerstad et al.

**Supplemental Table 1** P-values for Spearman correlation coefficients between non-fasting total alkylresorcinols and alkylresorcinol homologues, gluten, gluten-containing grains and dietary fiber in children at the age of 18 months. Dietary intake was estimated by 3-day food records.

|  | **Gluten g/day** | **Gluten  g/1000 kcal/day** | **Gluten-containing grains**  **g/day** | **Wheat**  **g/day** | **Rye**  **g/day** | **Barley**  **g/day** | **Dietary fiber**  **g/day** |
| --- | --- | --- | --- | --- | --- | --- | --- |
| **Total cohort (n=65)** |  |  |  |  |  |  |  |
| Alkylresorcinols, total | <0.001 | <0.001 | <0.001 | <0.001 | <0.001 | 0.057 | 0.899 |
| C17 | <0.001 | <0.001 | <0.001 | <0.001 | <0.001 | 0.012 | 0.309 |
| C19 | <0.001 | <0.001 | <0.001 | <0.001 | <0.001 | 0.049 | 0.811 |
| C21 | <0.001 | <0.001 | <0.001 | <0.001 | <0.001 | 0.070 | 0.981 |
| C23 | <0.001 | <0.001 | <0.001 | <0.001 | <0.001 | 0.070 | 0.903 |
| C25 | <0.001 | <0.001 | <0.001 | <0.001 | <0.001 | 0.038 | 0.681 |
| **Females (n=33)** |  |  |  |  |  |  |  |
| Alkylresorcinols, total | 0.002 | 0.001 | 0.003 | 0.001 | 0.105 | 0.541 | 0.580 |
| C17 | 0.002 | 0.001 | 0.003 | 0.002 | 0.024 | 0.161 | 0.180 |
| C19 | 0.001 | 0.001 | 0.002 | 0.001 | 0.054 | 0.470 | 0.550 |
| C21 | 0.001 | 0.001 | 0.002 | 0.001 | 0.133 | 0.639 | 0.789 |
| C23 | 0.004 | 0.002 | 0.006 | 0.003 | 0.213 | 0.577 | 0.556 |
| C25 | 0.006 | 0.004 | 0.006 | 0.003 | 0.121 | 0.289 | 0.395 |
| **Males (n=32)** |  |  |  |  |  |  |  |
| Alkylresorcinols, total | <0.001 | <0.001 | <0.001 | <0.001 | <0.001 | 0.062 | 0.902 |
| C17 | <0.001 | <0.001 | <0.001 | <0.001 | <0.001 | 0.053 | 0.717 |
| C19 | <0.001 | <0.001 | <0.001 | <0.001 | <0.001 | 0.055 | 0.997 |
| C21 | <0.001 | <0.001 | <0.001 | <0.001 | <0.001 | 0.060 | 0.963 |
| C23 | <0.001 | <0.001 | <0.001 | <0.001 | <0.001 | 0.126 | 0.905 |
| C25 | <0.001 | <0.001 | <0.001 | <0.001 | <0.001 | 0.157 | 0.827 |

*Abbreviations: g; grams*
